# Anticonvulsant Effects of Synthetic *N*-(3-Methoxybenzyl)oleamide and *N*-(3-Methoxybenzyl)linoleamide Macamides: An In Silico and In Vivo Study

**DOI:** 10.3390/molecules30020333

**Published:** 2025-01-15

**Authors:** Karin Jannet Vera-López, Jorge Alberto Aguilar-Pineda, Rodrigo Martín Moscoso-Palacios, Gonzalo Davila-Del-Carpio, José Luis Manrique-Murillo, Badhin Gómez, Minerva González-Melchor, Rita Nieto-Montesinos

**Affiliations:** 1 Escuela Profesional de Farmacía y Bioquímica, Universidad Católica de Santa María, Urb. San José s/n, Umacollo, Arequipa 04000, Peru; kvera@ucsm.edu.pe (K.J.V.-L.); rodrigo.moscoso@ucsm.edu.pe (R.M.M.-P.); gdavilad@ucsm.edu.pe (G.D.-D.-C.); bgomez@ucsm.edu.pe (B.G.); 2 Instituto de Física “Luis Rivera Terrazas”, Benemérita Universidad Autónoma de Puebla, Av. San Claudio, Cd. Universitaria, Apdo. Postal J-48, Puebla 72570, Mexico; jaguilar@ifuap.buap.mx; 3 Centro de Investigación en Ingeniería Molecular—CIIM, Universidad Católica de Santa María, Urb. San José s/n—Umacollo, Arequipa 04000, Peru; joseluismanriquemurillo@gmail.com

**Keywords:** macamides, epilepsy, neuroprotective effects, rFAAH, molecular dynamics

## Abstract

Epilepsy is a chronic neurological disorder that affects nearly 50 million people worldwide. Experimental evidence suggests that epileptic neurons are linked to the endocannabinoid system and that inhibition of the FAAH enzyme could have neuroprotective effects by increasing the levels of endogenous endocannabinoid anandamide. In this context, the use of macamides as therapeutic agents in neurological diseases has increased in recent years. With a similar structure to anandamide, several theories point to the FAAH–macamide interaction as a possible cause of FAAH enzymatic inhibition. In this work, we used in silico and in vivo techniques to analyze the potential therapeutic effect of three synthetic macamides in the treatment of epilepsy: *N*-3-methoxybenzyl-oleamide (3-MBO), *N*-3-methoxybenzyl-linoleamide (3-MBL), and *N*-3-methoxybenzyl-linolenamide (3-MBN). In the first stage, an in silico analysis was conducted to explore the energetic affinity of these macamides with rFAAH and their potential inhibitory effect. MD simulations, molecular docking, and MM/PBSA calculations were used for these purposes. Based on our results, we selected the two best macamides and performed an in vivo study to analyze their therapeutic effect in male Sprague Dawley rat models. Rats were subjected to an in vivo induction of epileptic status by the intraperitoneal injection of pilocarpine and analyzed according to the Racine scale. In silico results showed an energetic affinity of three macamides and a possible “plugging” effect of the membrane access channel to the active site as a potential cause of FAAH inhibition. On the other hand, the in vivo results showed an anticonvulsant effect of both macamides, with 3-MBL being the most active, resulting in a higher survival probability in the rats. This work represents one of the first studies on the use of macamides for the treatment of epilepsy.

## 1. Introduction

*Lepidium meyenii* (Maca) is a root that grows in central Peru, way up on the high plateaus of the Andes Mountains. Experimental and clinical studies have shown that, besides its nutritional value, Maca contains various bioactive components that give it therapeutic and medicinal properties [1,2]. For example, the inclusion of Maca in the human diet is used as an aphrodisiac to improve sexual performance and libido in men and women [3]. Studies carried out on male rats and mice with erectile dysfunction showed that the oral administration of extracts from this root improved sexual function and decreased the latency period of erection in all treated animals [4,5]. In addition, it increases the amount of sperm, modulates the symptoms of menopause [3], increases physical resistance [6], and exerts neuroprotective effects [7,8]. Among the various components present in Maca, the secondary metabolites known as macamides are considered the main ones responsible for these properties [9,10,11,12]. To date, more than 26 macamides have been identified [13]. They have a benzylamine linked by an amide bond to a fatty acid, which can be a saturated chain of 8 carbons, a saturated chain of between 15 and 18 carbons, or an unsaturated chain of 18 carbons. While in some macamides the fatty acid chain is modified to contain ketone functions [14,15], other macamides possess a methoxy group in the metaposition of the benzyl ring [14].

The endocannabinoid system (ECS), through the CB1 receptor, is involved in various brain events such as pain perception, the processing of reward and emotions, and learning and memory, as well as in providing neuroprotection in traumatic brain injury and neurodegeneration [16,17,18,19,20]. The CB1 receptor is bound by anandamide (*N*-arachidonylethanolamide), an endogenous ligand [21,22,23]. Nonetheless, a membrane-bound homodimeric allosteric enzyme, fatty acid amide hydrolase (FAAH) [24,25], down-modulates anandamide content and activity [26,27,28]. Interestingly, non-polar Maca extracts and macamides have demonstrated a pivotal neuroprotective effect via the endocannabinoid system [7,10]. Whereas some studies suggest that macamides inhibit FAAH activity and thereby enhance anandamide amounts [14,29], some others indicate that macamides could bind directly to the CB1 receptor [11,30].

Furthermore, in vivo models of epilepsy and patients with temporal lobe epilepsy displayed alterations in the endocannabinoid system [31,32,33] and decreased levels of anandamide in samples of cerebrospinal fluid [34]. In addition, it has been shown that the inhibition of FAAH [33,35] and modulation of anandamide signaling represent alternatives in treating epilepsy [36]. Hence, macamides could allow significant amounts of anandamide in cannabinergic synapses, a correct endocannabinoid system activity, and, consequently, an anticonvulsant effect.

In rats and humans, the FAAH enzyme is abundantly expressed in the brain and nervous system [37,38] as well as tissues like those in the kidney [39,40], female reproductive system [41,42], bladder [43], and liver [44], among others. Its activity is associated with multiple diseases and pathologies [37,41,45,46]. Due to its importance in modulating the ECS, FAAH is considered a therapeutic target. Its enzymatic mechanism has been widely studied, experimentally and with in silico techniques [25,27,28,41,45].

Structurally, rat FAAH (rFAAH) is a homodimeric enzyme with 579 residues per monomer and a molar mass close to 63 kDa [37]. Located in the cytoplasmic region of the cell, this enzyme is anchored to the cell membrane by two regions comprising residues T9-L33. Crystallographic studies have shown that for a substrate to reach the catalytic site (CT), formed by residues K142, S217, and S241, it must pass through two regions: the membrane access channel (MAC) and the acyl chain binding channel (ACB) [27]. These cavities are bordered by the residues D403, I407, R486, and I530 (MAC) and K335, E373, R428, and F527 (ACB). Some authors mention a MAC/ACB transition region composed of the residues F381, F432, and W531 [27]. Furthermore, it has two additional key sites: an oxyanion (OH) hole formed by the residues I238, G239, and G240 and the cytosolic port through which hydrolyzed substrates exit.

There are several macamides whose therapeutic effects in neurological disorders are being evaluated. However, three of them have stood out for their neuroprotective activity: *N*-(3-methoxybenzyl)oleamide (MAC 18:1), *N*-(3-methoxybenzyl)linoleamide (MAC 18:2), and *N*-(3-methoxybenzyl)linolenamide (MAC 18:3) [11,12]. This protective effect in neurotoxic environments and their structural similarity with anandamide make these molecules promising drugs in the treatment of the pathophysiology of neurological diseases, including epilepsy. Faced with this challenge, we have set the main objective of this work to evaluate whether these three macamides show neuroprotective activity against this neurological disorder. To achieve this goal, we performed a molecular analysis of one of the possible mechanisms in the anticonvulsant action of macamides: the inhibition of the catalytic activity of the enzyme rFAAH, which is key to the degradation of anandamide. Using various in silico techniques (MD simulations, molecular docking, interaction energies, among others), we evaluated the structural and energetic affinity of the macamide—rFAAH complexes. The in silico analyses confirmed a high affinity and a “plugging” effect of the macamides on the access channel to the membrane of rFAAH. These results support the idea that the therapeutic effect is due to the inhibition of rFAAH. Given these results, in vivo tests were performed to evaluate the protective effect of MAC 18:1 and MAC 18:2 against status epilepticus induced by the administration of pilocarpine. The analyses showed that these two macamides have an anticonvulsant effect in addition to reducing mortality in rat models. Given these promising results, we hope these findings will contribute to using and researching macamides as potential drugs in treating epilepsy.

## 2. Results and Discussion

### 2.1. In Silico Results

To facilitate reading and understanding in the discussion of the in silico results, simplified names and color codes are used to represent the different macamides. Thus, for *N*-(3-methoxybenzyl)oleamide, the name 3-MBO and the color gray are used in graphs and figures; for *N*-(3-methoxybenzyl)linoleamide, 3-MBL and the color green are used, and for *N*-(3-methoxybenzyl)linolenamide, the name 3-MBN and the color purple are used.

#### 2.1.1. Structure and ADMET Analysis of Macamides

**Figure 1:**
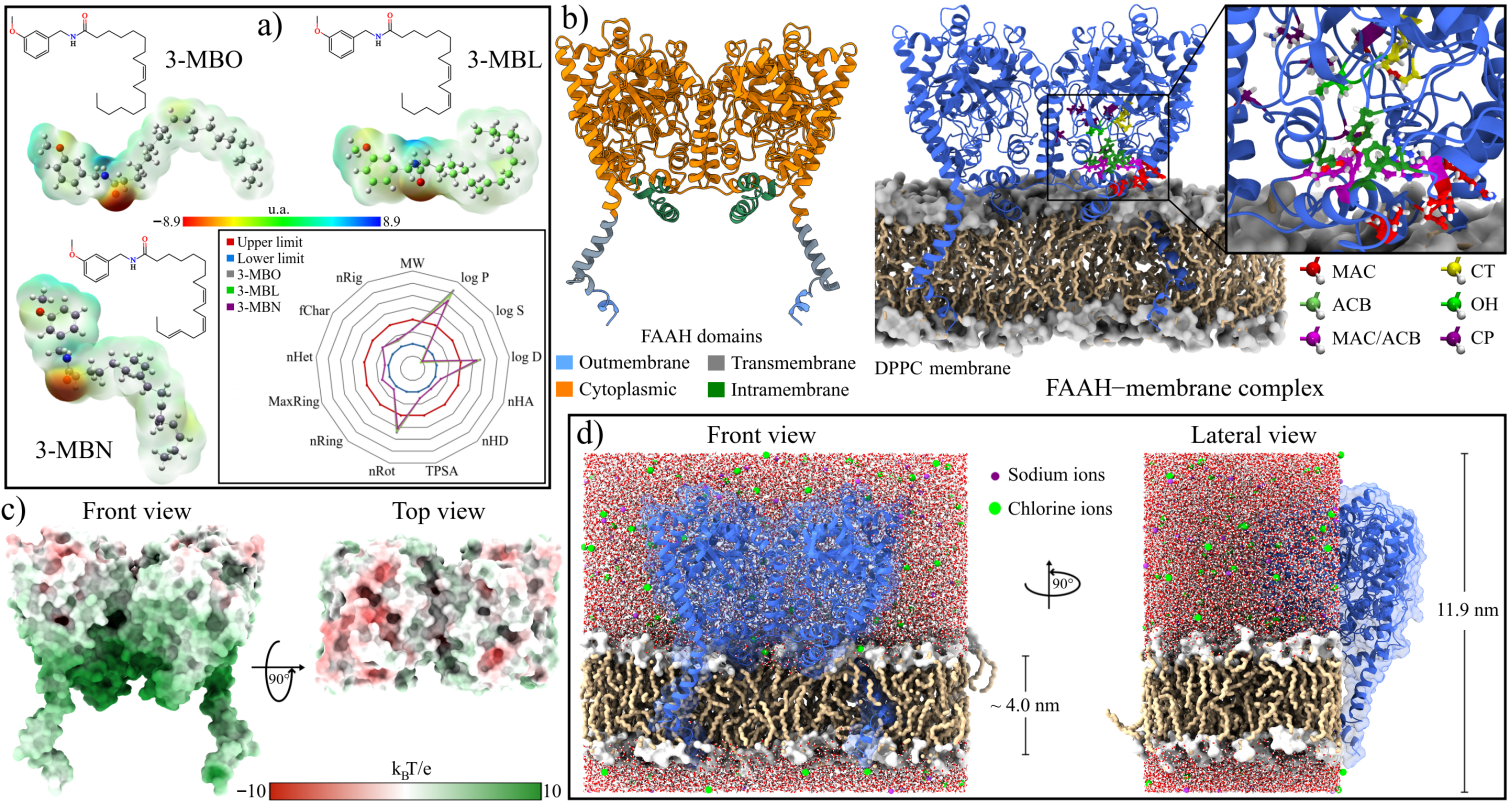
Analysis of the initial structures used in the MD simulations. (**a**) 2D and 3D structures of the three macamides used in this work. Semi-empirical quantum calculations were used to obtain the electrostatic potential surfaces. A red color represents regions of high electron density, and blue represents regions of low electron density. The green color indicates nonpolar regions. (**b**) The main domains of the dimeric rat fatty-acid amide hydrolase 1 (rFAAH) enzyme and construction of the rFAAH–membrane complex using the DPPC lipid model. (**c**) The electrostatic potential surface of rFAAH using the APBS approximation. Red represents nucleophilic regions, white represents nonpolar regions, and blue represents electrophilic regions. (**d**) An all-component system view for the MD simulations of the rFAAH enzyme. The height of the simulation cells (*z*-axis) was set so that there were no spurious interactions with the periodic images.

Structurally, like all macamides, they consist of an *N*-benzyl group attached to a fatty acid chain: oleic (3-MBO), linoleic (3-MBL), and linolenic (3-MBN). On the aliphatic chain, 3-MBO has a double bond in position C9, 3-MBL has two double bonds (C9 and C12), and 3-MBN has three double bonds (C9, C12, and C15). According to electrostatic potential (ESP) surface analysis, their structures have an electrophilic region around the nitrogen atom and one with a high electron density on the oxygen atom located in the amide bond (Figure 1a). In all three macamides, the double bonds give a weak nucleophilic character to the aliphatic chain. Furthermore, these macamides contain a methoxy group attached to the benzyl ring in position 3. As reported by Gugnani et al., this functional group could confer neuroprotective activity to these macamides by interacting with electron acceptor species [11].

As a first step to the in silico analyses, the physicochemical, medicinal–chemical, and ADMET properties of the three macamides were evaluated using the ADMETlab 3.0 server [47]. The results are shown in the inset in Figure 1a (physicochemical properties) and Table 1. As seen in the radar graph (Figure 1a), most of the normalized values of the three macamides were within the optimal parameters. The out-of-range values of logP, logD, and logS indicated greater solubility in non-polar environments, a behavior expected due to the aliphatic chain of their structures. However, this would be advantageous given the low-electrostatic environment in which they interacted with the rFAAH enzyme. The values for the medicinal–chemical parameters were optimal, as they were considered easy to design and synthesize (SAscore and GASA score). Although the QED parameter values indicated that they are undesirable drugs compared to other structures (op: >0.67), this could be due to the novelty of these molecules and the lack of research on their pharmacological properties. Moreover, macamides exhibit good absorption or permeability (Lipinski rule) and ADMET profiles (GSK rule and Golden Triangle). In addition, the three macamides obtained excellent evaluations in absorption properties: high permeability in Caco-2, MDCK cells, and the parallel artificial membrane permeability assay (PAMPA). Only 3-MBO was shown to be partially inhibitory to P-glycoprotein, and all three were not Pgp substrates and had high absorption in the human intestine absorption (HIA).

The properties not well evaluated were the distribution, excretion, and metabolism parameters. Regarding the distribution of macamides in the body’s tissues, only in the penetration of the blood–brain barrier (BBB) properties were values close to optimal obtained (op: ∼0). However, this property is fundamental in the study presented since different studies have shown that alterations in or the poor functioning of the BBB are involved in the pathogenesis and progression of several neurological diseases [48,49,50]. In the case of excretion properties, the prediction shows that the three macamides would have moderate plasma drug clearance rates (5–15 mL/min/kg) as well as ultra-short half-lives (<1 h). In the trials performed in the present work, the doses were administered intravenously according to the pilot results. In the evaluation of human cytochromes P450 (CYPs), a superfamily of enzymes that catalyze the metabolism of a wide range of endogenous and xenobiotic compounds [51], the results were that all three macamides were inhibitors of the CYP1A2, CYP2C19, CYP3A4 enzymes and substrates of CYP2C9 and CYP2D6. They were not substrates of the CYP1A2 and CYP3A4 enzymes; in particular, 3-MBO was not a substrate of CYP2C19. For 3-MBL and 3-MBN, they were not inhibitors of CYP2D6. CYP enzymes, specifically IA2, 2C9, 2C19, 2D6, and 3A4, are responsible for approximately 90% of oxidative metabolic reactions [51,52].

Finally, the three macamides showed promising results in toxicological prediction. They did not present a probability of liver damage or acute oral toxicity in the rats, are within the maximum daily dose (recommended) of the FDA, are not carcinogenic, and do not produce hepatotoxicity in humans. On the other hand, they presented toxicity for skin sensitization and eye irritation. In addition, macamide 3-MBN obtained adverse scores for eye corrosion and respiratory toxicity parameters.

#### 2.1.2. Initial Structures and Construction of Molecular Systems

Different in silico analyses were performed to corroborate the experimental results obtained. Since experimental evidence suggests that the observed therapeutic action of the macamides 3-MBO and 3-MBL could be due to their ability to inhibit the hydrolyzing action of FAAH on endocannabinoids [2,14], we constructed rFAAH–macamide complexes. For this, highly conserved pocket search calculations were performed on the rFAAH structure. Once these interaction sites were located, molecular docking analyses were performed using minimum energy structures obtained in free energy landscape (FEL) analysis. The complexes with the best energetic evaluation were subjected to molecular dynamics simulations in order to evaluate their structural and energetic affinity.

As a first step for analyzing these complexes, two MD simulations of the structure of rFAAH embedded in a DPPC-type lipid membrane were performed. To do this, two independent rFAAH–membrane structures were built, taking as structural domains those reported in the UniProt database (Figure 1b, left panel) [53,54]. The structure formed two lobes exposed in the cytoplasmic zone formed by the sequences R30-D403 and N434-S579 of each monomer. Special care was taken to ensure that the transmembrane (T9-L29) and intramembrane (L404-L433) regions of the enzyme were part of the lipid matrix. In Figure 1b, the right panel and inset show the regions involved in the catalytic activity of the enzyme that have been reported in numerous studies.

Electrostatic potential analysis of the initial structure shows that the surfaces of rFAAH are composed of two distinct regions (Figure 1c and Figure S1). While in the cytoplasm-exposed region, hydrophobic zones and nucleophilic areas are observed, especially in the cavities of the enzyme, at the interface with the membrane, the electrophilic character dominates. In particular, it can be noted that the cavity formed by the MAC domain, as well as the residues F432 and W531, present predominantly electrophilic zones (Figure S1a,c). These electrophilic characteristics could represent anchoring points with the three macamides due to their regions of high electron density on the oxygens of the methoxy and carbonyl groups and, to a lesser extent, on the double bonds (Figure 1a). Figure 1d shows the complete system used in the MD simulations. The initial thickness of the lipid bilayer corresponds to that obtained in the equilibrium stage of the system.

#### 2.1.3. MD Trajectories Analysis Showed Three Regions with High Conserved Pocket Density and a New Nucleophilic Cavity on the rFAAH Structure

**Figure 2:**
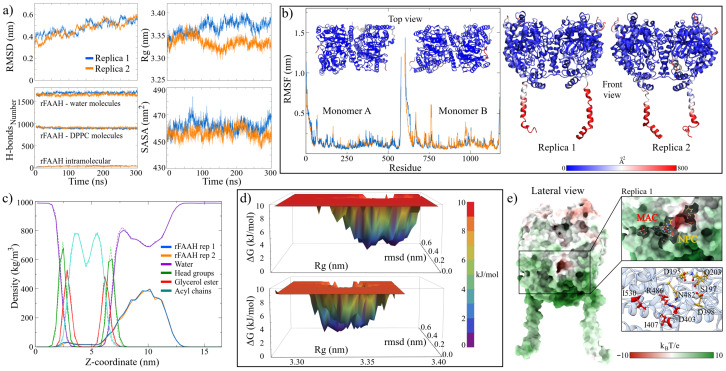
Results of MD simulations of the rFAAH enzyme embedded in a DPCC–lipid bilayer. (**a**) Stability indicators used to determine the convergence in the trajectories. (**b**) Analysis of the per-residue fluctuation of the rFAAH structures. The b-factor values were mapped onto the final configurations of the enzyme. The inset figures show the b-factors from a top view. (**c**) Partial densities of the different components of the rFAAH–membrane complex. The blue and orange lines correspond to the densities of the rFAAH enzyme in both replicates. The purple, green, red, and light blue lines indicate the densities of water, head groups, glycerol ester, and acyl chains. The dotted lines correspond to the values obtained in the simulation of the second replicate. (**d**) FEL analysis of the simulations. (**e**) A side view of the electrostatic potential surfaces of the final configurations in both simulations. The marked region corresponds to the entrance of the MA channel and a conserved pocket with nucleophilic features in both simulations. The residues involved in this pocket are D195, S197, Q203, D398, and N482.

Table 2 and Figure 2a show the stability indicators used to measure the structural convergence of the two replicas of the rFAAH enzyme. The values were obtained in the last 100 ns of the MD trajectories, while the graphs show the entire trajectory. Similarities can be observed in both the values and their fluctuations in both simulations. In general, the structure of rFAAH was stable under the simulation conditions, which has been corroborated in other studies [45,46]. The most notable difference was observed in the number of hydrogen bonds (HBs) of the measured interactions. In the second replica, the intramolecular HBs were more numerous on average, decreasing interactions with the solvent molecules (2.0%) and the membrane (−19.0%). On the other hand, the membrane exhibited a greater interaction with the solvent, increasing the number of HBs by around 1.9% compared to the value obtained in the first replica.

This structural stability can be appreciated by structurally aligning the final configurations with the initial ones (Figure S2). With RMSD values of 5.62 and 5.78 Å for both replicas, a high conservation of the extramembrane domains is observed. Part of this structural difference lies in the positions of the transmembrane and N-terminal domains. These domains acquired closed conformations concerning the frontal plane and open to the sagittal plane. This mobility is reflected in the fluctuation per residue analysis represented in the RMSF plots and the b-factor values mapped onto the final structures of the two replicates (Figure 2b). Other zones with high fluctuations were the R63-L69 and P574-S579 moieties. On the other hand, the structural integrity of the membrane was verified by analyzing the partial densities of its components. Figure 2c shows the superimposed density curves of both replicas; notice that they are preserved in both simulations. The membrane thickness, measured by the highest density peaks of the head groups, were 4.26 and 4.20 nm for replicas 1 and 2, respectively. These values agree with the experimental data at 309.15 K (3.6–4.4 nm) [55] and other in silico studies [56,57,58].

In order to determine the structure for molecular docking calculations, a free energy landscape (FEL) analysis of the enzyme configurational microstates along the MD trajectories was performed. Figure 2d shows the graphs of this analysis, allowing us to observe a considerable amount of low-energy microstates in both simulations. This distribution of metastable configurations exhibits the structural flexibility of rFAAH despite its high stability [46]. In the analysis of minimum energy configurations, three were found for the first replica at 39.2, 280.0, and 282.2 ns, with maximum energy states of 9.76 kJ/mol (Figure S3a). On the other hand, for the second replica, four configurations were obtained (t = 127.2, 151.4, 181.2, and 212.2 ns) with maximum energy microstates of 9.18 kJ/mol (Figure S3b). Based on these results, the structure at 282.2 ns obtained from the first simulation was chosen.

It is noteworthy that in the structural analysis, when recalculating the electrostatic potentials of the final configurations, the formation of a pocket with nucleophilic characteristics close to the MAC was observed (Figure 2e). This nucleophilic cavity (NPC) was also observed in the second replica and the minimum energy structures (Figure S4). This NPC was formed by the electron donor contribution of the oxygen atoms of the D195, S197, Q203, D398, and N482 residues (inset Figure 2e). Due to its location, this site could serve as an anchoring point for electron acceptor molecules or as an allosteric site in the catalytic activity of rFAAH. However, more advanced studies are required to determine its function or its conservation at more extended times in MD simulations.

**Figure 3:**
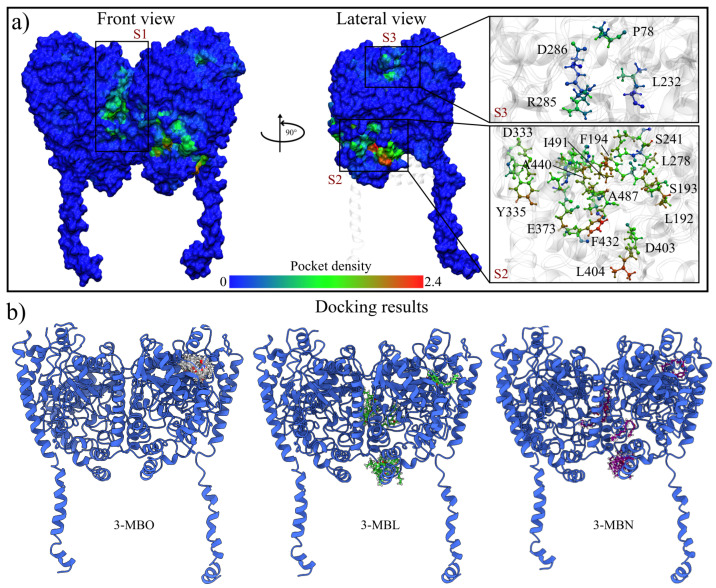
High-probability sites of molecular interaction. (**a**) The conservation of pockets obtained through the complete MD trajectories. The probability of being part of a conserved pocket is indicated by colors, with blue indicating regions with a low probability, green areas indicating a medium probability, and red indicating a high likelihood. (**b**) rFAAH–macamide interaction complexes. The different poses correspond to the ten best energetically favorable solutions.

For this work, the analysis of macamide interactions at the catalytic site of the rFAAH structure was not aimed at. Instead, interactions on the enzyme surface were analyzed to determine possible allosteric sites where macamides could interact. For this, two different methodologies were used: calculations of searching for highly conserved pockets in MD trajectories using the MDPocket server [59,60] and molecular docking using the AutoDock Vina program [61,62]. The results of the pocket analysis show three regions with a high probability of being interaction sites (Figure 3a). Two are the canonical sites already known: the domains responsible for the hydrolysis of endocannabinoids and the one corresponding to the cytosolic port, located at the interface between the two monomers. Similar results were reported by Morgillo et al., who divided these regions into seven main pockets [46]. A third highly conserved pocket was located on the surface exposed to the cytoplasm, formed by the residues Q577, P578, and S579 of the C-terminal domain and the residues P78, L79, and L80.

Similarly, molecular docking results showed that rFAAH–macamide complexes with the highest energetic affinity interacted in the high-conservation pocket regions. Figure 3b shows the top ten energetically favored solutions of each macamide studied. It can be observed that for the case of 3-MBO, all ten solutions were located in the S3 site. Meanwhile, the solutions for the macamides 3-MBL and 3-MBN were located in all three sites.

#### 2.1.4. BFE Analysis Showed That 3-MBO Macamide Was Strongly Binding with rFAAH

As shown by the previous analyses, there are three surface regions in rFAAH with a high probability of interaction with macamides (Figure 3). However, in this study, we focused the interaction analysis only on the S2 and S3 sites. Site S2 was chosen because it is located in the region that includes residues that are part of the membrane access channel (bottom inset of Figure 3a). Regarding the S3 site, it was the only one in which the three macamides presented favorable interactions among the ten best energetically evaluated complexes, according to the molecular docking results. Since the S1 site corresponds to the region where the cytosolic port is located, which directs the hydrolyzed endocannabinoids towards the cytoplasm [45], we decided to analyze it in future work.

**Figure 4:**
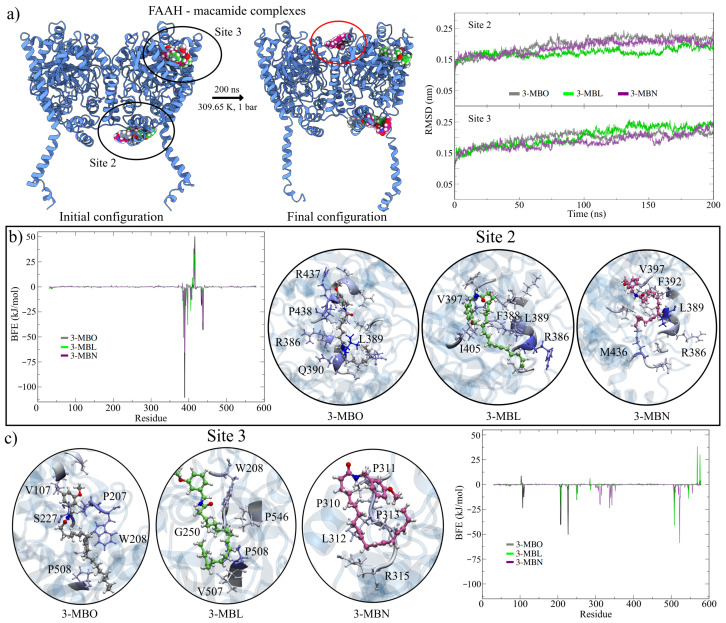
Binding free energy (BFE) analysis. (**a**) Initial and final complexes in the MD simulations. The initial position for the macamide 3-MBO at the S2 interaction site was obtained from the best-ranked solution of the complexes obtained from the docking results. In the remaining cases, the best-ranked solution was always taken. The final position of macamide 3-MBN at site 3 shows a shift towards site 1 (red circle), while the remaining ones were conserved in the interaction sites. The RMSD graph shows the structural evolution of rFAAH through the MD trajectories. (**b**,**c**) Zoom details of the macamide interactions at the S2 and S3 sites. Only the five residues with the highest contribution to the binding energies are shown. The graphs show the energies per residue for each interaction analyzed.

The complexes chosen for the MD simulations were those that obtained the highest interaction energy from the docking calculations at sites S2 and S3. The values of the stability indicators are given in Table 2 and show that the rFAAH–macamide systems were very stable. Figure 4a, in the left panel, shows the initial and final positions of the three macamides at the sites analyzed, and Figure S5 shows the interactions with the rFAAH residues. For site S2, a slight displacement of the macamides from their original position towards the MA channel is observed without interacting with it. In the RMSD graphs (Figure 4a, right panel), it can be seen that the simulations converged after 150 ns of simulation. On the other hand, at site S3, the macamides 3-MBO and 3-MBL remained in the molecular pocket. Interestingly, it is observed that the macamide 3-MBN left the pocket and moved towards site S1.

The energetic affinity of macamides for the S2 and S3 sites was determined by performing binding free energy (BFE) analyses in the MM/PBSA approximation with the g_mmpbsa v1.6 software [63]. Only the last 50 ns of the MD trajectories were analyzed for these calculations, where the systems showed the highest structural stability. Table 3 shows the results obtained for each energy term given by Equation (1).

The results show a high energetic affinity of the macamides at the interaction sites studied. However, the energies obtained at the S2 site were, on average, almost 50% higher than those obtained at the S3 site. Furthermore, as mentioned above, 3-MBN was non-affinity to S3 and migrated towards the S1 site. This result could indicate that if there were interactions between the macamides and rFAAH, they would have a greater probability of occurring at the S2 site. Furthermore, the simulations show that, although there was a shift, the interactions with the MAC residues were not significant, remaining in the interaction pocket. Based on these results, a possible inhibitory mechanism of macamides could be due to a “plug” effect on this channel, which would prevent the entry of endocannabinoids to the catalytic site.

On the other hand, of the three macamides, 3-MBO showed the highest energetic affinity at both interaction sites. These results disagree with those found in our experimental studies, where the greatest therapeutic effect was with macamide 3-MBL. However, the energies are close, with differences less than 13.63 kJ/mol (∼3.26 kcal/mol). Figure 4b,c shows the energy analysis per residue of the different complexes, while Table S1 lists the ten residues with the highest energetic contribution to the BFE. Among these residues with the highest contribution, R386, L389, F392, V397, and M436 were involved in the interaction with the three macamides at the S2 site, while the residues W208, G250, P508, and P546 were for the S3 site.

Finally, a new pocket conservation analysis was performed, taking the MD trajectories of the rFAAH–macamide simulations in order to qualify the effect of the macamide interaction. For this, the probability of being a hotspot was calculated only for the residues involved in the enzyme’s catalytic activity. This calculation was performed on each monomer of the replicas without the presence of macamide and on the monomer that interacted with them (Table 4). The results were significant. As expected, there was a structural effect on the conformation of rFAAH due to macamides in the interaction sites. However, in the S2 site, a decrease in the values of this parameter was observed in the residues that formed the MAC and MAC/ACB domains. These findings would further support our idea of a possible “plug” effect of macamides on the active site of rFAAH. This plug effect was more evident with the macamide 3-MBL, which may partly explain why it had a more significant therapeutic effect than 3-MBO.

On the other hand, the effect of macamides on the S3 site was not noticeable for the domains analyzed. This was expected due to the distance to the catalytic site. However, a decrease in the probability values was observed in the residues Y335 and E373 of the ACB domain and I530 of the MAC. These findings suggest that the effect of macamides on this site was allosteric; however, further analysis is necessary to elucidate the possible function of this pocket.

### 2.2. In Vivo Results

**Figure 5:**
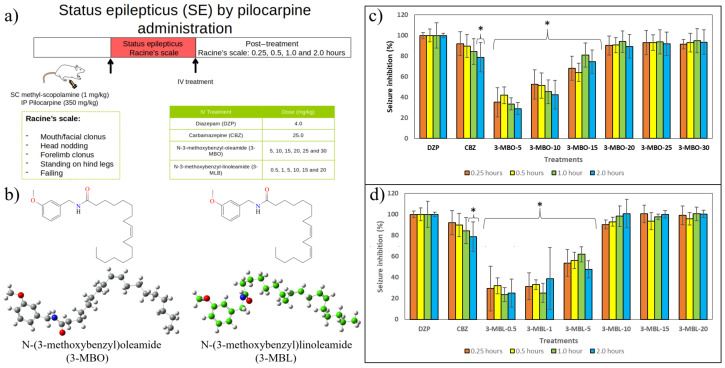
Experimental results. (**a**) Flowchart of status epilepticus (SE) by pilocarpine administration. (**b**) Two- and three-dimensional structures of *N*-(3-methoxybenzyl)oleamide and *N*-(3-methoxybenzyl)linoleamide. Seizure inhibition (%) on pilocarpine-induced status epilepticus after administration of following: (**c**) diazepam (DZP), carbamazepine (CBZ), and *N*-(3-methoxybenzyl)oleamide at 5.0 mg/kg (3-MBO-5), 10.0 mg/kg (3-MBO-10), 15.0 mg/kg (3-MBO-15), 20.0 mg/kg (3-MBO-20), 25.0 mg/kg (3-MBO-25), and 30.0 mg/kg (3-MBO-30); and (**d**) diazepam (DZP), carbamazepine (CBZ), and *N*-(3-methoxybenzyl)linoleamide at 0.5 mg/kg (3-MBL-0.5), 1.0 mg/kg (3-MBL-1), 5.0 mg/kg (3-MBL-5), 10.0 mg/kg (3-MBL-10), 15.0 mg/kg (3-MBL-15), and 20.0 mg/kg (3-MBL-20). Percentage of seizure inhibition is expressed as mean ± S.D. Bars represent standard deviation. *n* = 6. * Anova test = significantly different to response displayed by group that received diazepam at 4.0 mg/kg.

In this study, status epilepticus was induced by intraperitoneal pilocarpine administration at a dose of 350 mg/kg. The pilocarpine dose was chosen based on the latency and mortality rate in the pilot study, which is in accordance with previous studies [64]. Status epilepticus began within one hour of pilocarpine injection but was preceded by intermittent seizures. During the status epilepticus, the animals showed mouth/facial clonus, head nodding, forelimb clonus, standing on hind legs, and falling (Figure 5a), according to Racine’s scale.

Diazepam is one of the main pharmacotherapies used to stop status epilepticus and used as a control to assess the anticonvulsant properties of new entities [64]. Herein, diazepam was administered at a dose of 4.0 mg/kg by an intravenous route as previously reported [65]. The intravenous route is the most efficient means of delivering drugs to experimentation animals [66]. Drugs administered intravenously can directly reach blood circulation and thereby assure 100% bioavailability because they are not subject to absorption or first-pass metabolism. In that sense, drugs can successfully reach their target site [67,68]. Nevertheless, future studies could assess the anticonvulsant properties of macamides using other administration routes, especially the oral route, which is convenient, cost-effective, and the most intended in the late phases of drug development [68].

Given that all treatments were administered intravenously, the onset of seizure inhibition was observed in the five minutes post-dosing of diazepam, carbamazepine, 3-MBO at doses higher than 15 mg/kg, and 3-MBL at doses higher than 10 mg/kg (Figure 5c). Figure 5c shows the effect of 3-MBO on pilocarpine-induced status epilepticus. Response to diazepam was considered 100.0% at 0.25, 0.5, 1.0, and 2.0 h post-treatment.

Compared to diazepam, carbamazepine reduced signs of status epilepticus by 92.13%, 89.85%, and 84.45% at 0.25, 0.5, and 1.0 h, respectively. However, at 2.0 h, the carbamazepine effect decreased significantly to 78.83%, related to diazepam. 3-MBO at 5.0 and 10.0 mg/kg could not mitigate signs of status epilepticus. The same compound at 15.0 mg/kg showed a mild anticonvulsant effect; however, at doses of 20.0, 25.0, and 30.0 mg/kg, it succeed in relieving the number and severity of seizures, displaying a response superior to 89.0%, compared to diazepam’s effect.

Figure 5d shows the effect of 3-MBL on pilocarpine-induced status epilepticus. Herein, as in the previous picture, response to diazepam was also considered 100.0% until 2.0 h post-treatment. 3-MBL at 0.5, 1.0, and 5.0 mg/kg obtained a significantly lower response compared to diazepam and carbamazepine 0.25, 0.5, 1.0, and 2.0 h after their administration. Nonetheless, 3-MBL at 10.0, 15.0, and 20.0 mg/kg exhibited similar anticonvulsant effects to diazepam, showing a response higher than 90.0% in the four time points.

**Figure 6:**
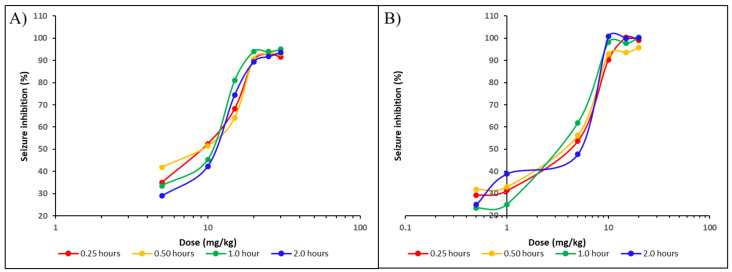
Dose–response curves of 3-MBO (**A**) and 3-MBL (**B**) on the percentage of seizure inhibition in rats that developed status epilepticus after intraperitoneal administration of pilocapine. The logarithmic graphical representation between the doses and anticonvulsant effects of both macamides were evaluated at four different times, 0.25 h (red), 0.50 h (yellow), 1.0 h (green) and 2.0 h (blue). *n* = 6. ED _50_ for 3-MBO ranged between 9.1 and 12.0 mg/kg and ED _50_ for 3-MBL ranged between 3.2 and 5.5 mg/kg.

The experimentally derived ${\mathrm{ED}}_{50}$ values for 3-MBO ranged from 9.1 to 12.0 mg/kg (Figure 6A), whereas those for 3-MBL ranged from 3.2 to 5.5 mg/kg (Figure 6B). These results indicate that 3-MBL would be more potent than 3-MBO.

Survival in every experimental group was followed for 48 h. Mortality in the group that received the vehicle began 3 h post-treatment and reached nearly 67.0% 48 h post-treatment. As expected, survival improved in the groups receiving controls, diazepam, and carbamazepine, reaching only a 17.0% mortality. In accordance with the anticonvulsant effect, doses above 20 mg/kg of 3-MBO displayed a similar survival path to carbamazepine. While 10 mg/kg of 3-MBL significantly reduced mortality, 15 and 20 mg/kg of 3-MBL successfully abolished it.

## 3. Materials and Methods

### 3.1. Computational Details

#### 3.1.1. rFAAH and Macamide Structures

The structure of the rat fatty acid amide hydrolase (rFAAH) monomer was retrieved from the AlphaFold server (AF-ID: AF-P97612-F1) [69]. To build the rFAAH homodimer, the crystal structure of the FAAH from *Rattus norvegicus* was used as a template (PDB ID: 4HBP) [70]. A double-layer model, with 256 dipalmitoylphosphatidylcholine (DPPC) molecules per layer, was used to simulate the lipid membrane, following the methodology proposed by Lemkul [71]. The transmembrane domains of the enzyme were embedded in the lipid matrix using the Inflategro methodology [72]. The simulation cells were adjusted to match the size of the membrane along the *x* and *y* axes. For the *z*-axis, the cell length was set so that the enzyme surface was at least 1.5 nm away from the cell edges to prevent any unwanted interactions caused by periodic boundary conditions. The systems were solvated using the TIP3P water model [73]. Some advantages of this model are its low computational cost and high self-diffusion, which could increase its efficiency by accelerating molecular simulations and increasing sampling in systems involving many atoms, such as biomolecular complexes [74]. In addition, this model has been used in other in silico studies involving the enzyme FAAH [75,76] and macamides [77]. To ensure that no water molecules were in the hydrophobic region of the lipid matrix (defined between the aliphatic chains and the ester group of the lipids), they were removed before the energy minimization process [71]. Finally, ${\mathrm{Cl}}^{-}$ and ${\mathrm{Na}}^{+}$ ions were introduced to balance the electrical charges and replicate the physiological conditions of all systems until reaching an ion concentration of 0.154 M.

Three macamide molecules were considered ligands in the rFAAH complexes: *N*-(3-methoxybenzyl)oleamide (MAC 18:1), *N*-(3-methoxybenzyl)linoleamide (MAC 18:2), and *N*-(3-methoxybenzyl)linolenamide (MAC 18:3) [11]. Three-dimensional structures were constructed using the GaussView v.6 software [78] and optimized by semi-empirical quantum calculations using the AM1 method [79] in the Gaussian 16 software package [80]. This semi-empirical method was used to accelerate the quantum calculation convergences and to obtain the input structures for the molecular docking analyses. The same level of theory was used to calculate their vibrational frequencies to ensure that the structures were in their lowest energy states. Using these optimized geometries and the CAM-B3LYP/TZVP level of theory [81,82], the partial atomic charges were calculated by Hirshfeld’s population analysis [83,84] in order to consider the electrostatic effect of macamides on the FAAH enzyme structure. These atomic charges were used to reparametrize their force fields obtained by the LigParGen server [85,86,87].

#### 3.1.2. MD Simulations

All MD simulations were performed using the GROMACS 2021 package [88,89]. Atomic interactions were calculated using the parameters of the OPLS force field in its all-atom version [90,91]. The lipid bilayer was constructed using the DPPC model with the interaction parameters proposed by Tieleman and Berendsen [92]. All systems were energy-minimized for 50,000 steps using the steepest descent algorithm. Then, two equilibrium simulations were performed, with the first using the NVT ensemble for 50 ps. The second simulation was performed using the NPT ensemble for two nanoseconds. In both simulations, the temperature of the system was 323.15 K, restricting the positions of the heavy atoms, both rFAAH enzymes and ligands. A V-rescale thermostat was used in the NVT simulations [93], while the Nosé–Hoover thermostat [94,95] and the Parrinello–Rahman barostat [96] with semiisotropic coupling were chosen for the NPT simulations. The compressibility factor was set at $4.5\times 10^{-5}$ L/bar. In the case of the system without the presence of the ligands, the positions and velocities in the NPT equilibrium simulations were saved for every nanosecond. These frames served as initial structures for the production simulations of the two replicas made of the rFAAH–membrane complexes.

All MD production simulations were conducted at a 309.65 K temperature and one pressure bar in the NPT ensemble. Although this temperature corresponds to the physiological temperature in humans, it is close to the average body temperature of adult rats (310.25 ± 0.2 K) [97]. For the ligand-free systems, the MD trajectories were run for 300 ns, while for the rFAAH–ligand systems, they were run for 200 ns. The production simulations were performed without position constraints and with semi-isotropic pressure coupling. The equations of motion were solved using the leap-frog integrator with a time step of 1 fs. Periodic boundary conditions (PBCs) were used in all directions. The particle mesh Ewald (PME) algorithm with cubic interpolation and a cutoff radius of 1.2 nm and spline of the order 4 was used for long-range interactions [98,99]. The bond lengths were kept rigid by using the LINCS algorithm [100,101]. The electrostatic interactions were treated with the Ewald sums with a $1\times 10^{-6}$ tolerance for the real space contribution. The real part of the Ewald summation and the Lennard–Jones interactions were truncated at 1.2 nm, including long-range corrections to the energy. The temperature was coupled to the Nosé–Hoover thermostat with a relaxation time constant of $\tau_{T}$ = 0.5 ps. The pressure was coupled to the Parrinello–Rahman barostat with a semi-isotropic pressure coupling and a relaxation time constant of $\tau_{P}$ = 2.0 ps. All trajectories were saved every ten ps.

#### 3.1.3. Highly Conserved Pocket Search

The highly conserved pockets and their probability values were calculated using the MDpocket server [59,60], which detects highly conserved void zones through the MD simulations. The MD trajectories were prepared considering only the rFAAH structures and saved them every five nanoseconds. Results were mapped on the atoms and surfaces of the rFAAH enzyme. To compare the probability values, these were normalized using the maximum value of the density of pockets.

#### 3.1.4. Molecular Docking Calculations

Rigid receptor docking calculations were performed using Autodock Vina 1.2.5 software [61,62]. The chosen host structure was the stabilized configuration of the rFAAH enzyme obtained from the MD trajectories without considering the lipid membrane. The optimized configurations of the three macamides obtained by semi-empirical quantum calculations were used as ligands. Docking was carried out using a search volume box with dimensions of 2 nm, additionally to the enzyme surface. Three thousand two hundred repetitions were performed for each macamide, obtaining ten binding modes for each event with a maximum energy difference of 3 kcal/mol and an exhaustiveness search of eight. The ten best energetically evaluated structures of each rFAAH–macamide complex were chosen for energetic refining using FireDock software (https://bioinfo3d.cs.tau.ac.il/FireDock/, accessed on 9 January 2025) [102]. The six best configurations were selected for MD simulations.

#### 3.1.5. Drug-Likeness Prediction

The ADMET parameters (absorption, distribution, metabolism, excretion, and toxicity), physicochemical properties, and pharmaceutical compatibility of macamides were evaluated using the ADMETlab 3.0 server [47].

#### 3.1.6. Binding Free Energy Using MM/PBSA Approximation

Binding free energies (BFEs) were used to evaluate the enzyme–macamide affinities. Molecular mechanics Poisson–Boltzmann surface area (MM/PBSA) calculations were performed using the g_mmpbsa methodology [63,103]. The energy contributions per residue were calculated to localize the main interactions and to assess the effect of each residue on the molecular complexes. The last 50 ns of the MD trajectories were analyzed at a regular interval of 0.1 ns to estimate the BFE ($\Delta G_{bind}$), which was calculated using the following equation:

(1) $$\Delta G_{bind}=G_{complex}-\left(G_{rFAAH_{enz}}+G_{macamide}\right)$$

where $G_{complex}$ is the total Gibbs free energy of the rFAAH–macamide complexes, and $G_{rFAAH_{enz}}$ and $G_{macamide}$ are the Gibbs free energies of isolated rFAAH enzyme and macamides, respectively. In addition, we used the bootstrap analysis to calculate the average binding energy, which is included in the g_mmpbsa tools. All calculations were obtained at 309.65 K, and default parameters were used to calculate molecular mechanics potential energy and solvation-free energy [63]. Finally, the BFE by residue was calculated by the following equation:

(2) $$\Delta G_{bind}^{res}=\Delta E_{MM}^{res}+G_{p}^{res}+G_{np}^{res}$$

This equation calculates the polar contribution to the BFE by solving the Poisson–Boltzmann (PB) equation [104]. At the same time, the non-polar term is estimated using the solvent-accessible surface area (SASA) approach [105,106].

#### 3.1.7. In Silico Structures and Data Analysis

The root mean square deviation (RMSD), root mean square fluctuation (RMSF), radii of gyration (RG), mean square displacement (MSD), hydrogen bond (HB), and solvent-accessible surface area (SASA) values were calculated using Gromacs tools. All analysis and structural properties were carried out in the last 100 ns of the MD trajectories. The structure analysis and figures were completed using visual molecular dynamics (VMD), UCSF Chimera v.1.14, and the UCSF Chimera X.1.6.1 software [107,108]. The XMGrace software (https://plasma-gate.weizmann.ac.il/Grace/, accessed on 9 January 2025) was used to plot graphs [109]. The APBS (Adaptive Poisson Boltzmann Surface) software v.1.4.1 was used to calculate the electrostatic potential (ESP) surfaces in the molecular mechanics framework [110], and the PQR input was created in the PDB2PQR server [111]. Free energy landscape (FEL) analysis was performed using the Gromacs gmx sham module and visualized with Mathematica 12.1 software [112].

### 3.2. In Vivo Assays

#### 3.2.1. Reagents

Pilocarpine, methyl-scopolamine, diazepam, carbamazepine, tetra glycol, and poly-ethyleneglycol 600 were obtained from Sigma Aldrich (St. Louis, MO, USA), and macamides were synthesized in the Organic Chemistry Laboratory at MCPHS University [14] (Boston, MA, USA).

#### 3.2.2. Drugs’ Preparation

The preparation of the drugs was carried out on the day of the experiment. Diazepam, carbamazepine, and both synthetic macamides were dissolved in tetra glycol at initial concentrations of 5.0 mg/mL, 80.0 mg/mL, and 30.0 mg/mL, respectively. Then, each compound was diluted in a mixture of polyethylene glycol 600 and purified water (1:3).

#### 3.2.3. Animals

The anticonvulsant effects of macamides were assessed in male Sprague Dawley rats at the Animal Facility of the Universidad Andina del Cusco. All animal experiments were carried out in accordance with the recommendations in the Guide for the Care and Use of Laboratory Animals (Institute of Laboratory Animal Resources, National Research Council, National Academy of Sciences, Washington, DC, USA). The Institutional Animal Care and Use Committee of the Universidad Peruana Cayetano Heredia approved the studies. All efforts were made to minimize animal suffering. The animals were maintained under a 12 h light/dark cycle and a temperature-controlled environment. Food and water were provided ad libitum. All the animals were allowed to acclimate for one week and were seven weeks old (250–300 g weight) at the time of the experiment.

#### 3.2.4. Induction of Status Epilepticus by Pilocarpine Administration

The Sprague Dawley rats were subjected to status epilepticus induction by a single intraperitoneal injection of pilocarpine, according to Turski et al. [113]. To counteract the peripheral cholinomimetic effects of pilocarpine, the animals received the subcutaneous administration of methyl-scopolamine at a dose of 1.0 mg/kg body weight. Thirty minutes later, the animals received the intraperitoneal administration of pilocarpine at a dose of 350 mg/kg. The pilocarpine dose was chosen based on the latency and mortality rate in the pilot study. Status epilepticus was defined as a phase of continuous seizures that lasted for at least 5 min or seizures that recurred at intervals shorter than one minute, thus displaying a persisting epileptiform condition [114]. Once the animals developed status epilepticus, they were observed to determine signs of seizure activity and severity according to Racine’s scale [115], which consists of the following stages: 1 = seizure consisted of immobility and occasional facial clonus; 2 = head nodding; 3 = bilateral forelimb clonus; 4 = rearing; 5 = rearing and falling.

#### 3.2.5. Experimental Procedure

The animals were randomly divided into fifteen experimental groups (*n* = 6). After one hour of being in status epilepticus, the different groups received the following treatments via the caudal vein by a single intravenous bolus [116]. Dosage was chosen based on our pilot study.

Group 1: a vehicle of diazepam, carbamazepine, and a synthetic macamide, which was a mixture of tetraglycol, polyethyleneglycol 600, and water (1:1:3). Group 2: diazepam, at a dose of 4 mg/kg body weight. Group 3: carbamazepine, at a 25 mg/kg body weight dose. Groups 4, 5, 6, 7, 8, and 9: *N*-(3-methoxybenzyl)oleamide at doses of 0.5, 1, 5, 10, 15, and 20 mg/kg body weight, respectively. Groups 10, 11, 12, 13, 14, and 15: *N*-(3-methoxybenzyl)linoleamide at doses of 5, 10, 15, 20, 25, and 30 mg/kg body weight, respectively. Ongoing behavior was observed and recorded for the following 48 h.

#### 3.2.6. Statistical Analysis

The statistical analysis was carried out using SigmaStat 3.5 software. Analyses of statistical significance between two groups were examined using Student’s *t*-test and between many groups by one-way analysis of variance (ANOVA) with the Holm–Sidak post hoc test. *p* < 0.05 was considered significant. Survival analysis was performed using the Kaplan–Meier method.

## 4. Conclusions

Epilepsy is one of the leading neurological disorders that affects around 0.6% of the world’s population, with 80% of patients from low-income strata. One of the most common symptoms is seizures, which is why great efforts are currently being made in the search for anticonvulsant drugs in order to improve the quality of life of patients. Various studies have shown that macamides have, among several properties, neuroprotective effects with very few adverse impacts and a low cost.

In this work, we addressed using three synthetic macamides to treat seizure symptoms: *N*-(3-methoxybenzyl)oleamide (N-MBO), *N*-(3-methoxybenzyl)linoleamide (3-MBL), and *N*-(3-methoxybenzyl)linolenamide (3-MBN). Our in silico results showed that the three macamides had a structural and energetic affinity with the enzyme rFAAH. These results support one of the hypotheses about the therapeutic effect of these macamides, which suggests that their interaction with the enzyme could inhibit its catalytic action on endocannabinoids. When performing in vivo tests of the two macamides with the highest affinity, 3-MBO at 20.0, 25.0, and 30.0 mg/kg and 3-MBL at 10.0, 15.0, and 20.0 mg/kg exhibited similar anticonvulsant effects to diazepam during status epilepticus induced by pilocarpine, thus improving survival. Nevertheless, 3-MBL may be more potent than 3-MBO.

Although there are limitations to this work, these promising results obtained in our analyses open up the possibility of using these macamides in anticonvulsant treatment in epileptic patients. New research is being conducted in our Latin American region, and we hope that this work can offer insight into developing new therapies to treat epilepsy.

## Figures and Tables

**Table 1:** ADMET prediction of the three macamides obtained using the ADMETlab 3.0 server.

Property	Parameter	Predicted Value ^a^			Property	Parameter	Predicted Value ^a^		
		3-MBO	3-MBL	3-MBN			3-MBO	3-MBL	3-MBN
Physicochemical	logS	−6.585	−6.623	−6.216	Metabolism	CYP1A2 inhibitor	+++	+++	+++
	logP	7.818	7.244	6.395		CYP1A2 substrate	- - -	- - -	- - -
	logD_7.4_	4.621	4.465	4.305		CYP2C19 inhibitor	+++	+++	+++
						CYP2C19 substrate	- - -	+++	++
Medicinal–Chemical	QED (>0.67)	0.206	0.224	0.243		CYP2C9 inhibitor	+	+++	++
	SAscore	Easy	Easy	Easy		CYP2C9 substrate	+++	+++	+++
	NPscore	−0.115	0.104	0.222		CYP2D6 inhibitor	+++	-	- - -
						CYP2D6 substrate	+++	+++	+++
Absorption	Caco-2 Perm. (>−5.15)	−5.016	−5.009	−4.982		CYP3A4 inhibitor	+++	+++	+++
	MDCK Perm.	0.0	0.0	0.0		CYP3A4 substrate	- - -	- - -	- - -
	PAMPA	- - -	- - -	- - -					
	Pgp-inhibitor	+	++	+++					
	Pgp-substrate	- - -	- - -	- - -	Toxicity	hERG Blockers	0.825	0.849	0.864
	HIA	- - -	- - -	- - -		DILI	0.04	0.002	0.0
						AMES Tox.	0.384	0.435	0.888
Distribution	PPB (<0.90)	0.987	0.984	0.983		Rat Oral Acute Tox.	0.071	0.028	0.032
	VDss (0.04–20)	0.595	0.792	0.707		FDAMDD	0.292	0.052	0.001
	BBB	0.081	0.067	0.174		Skin Sensitization	1.0	1.0	1.0
	Fu (%)	0.50	1.00	1.30		Carcinogencity	0.13	0.021	0.001
						Eye Corrosion	0.141	0.45	1.0
Excretion	CL	5.562	6.172	6.536		Eye Irritation	0.912	0.969	1.0
	$t_{1/2}$	0.386	0.191	0.158		Respiratory Tox.	0.545	0.56	0.944

^a^ Symbols used for the prediction probability values: 0–0.1 (- - -), 0.3–0.5 (-), 0.5–0.7 (+), 0.7–0.9 (++), and 0.9–1.0 (+++).

**Table 2:** Stability descriptors of the studied systems.

System	^a^ RMSD	^a^ Radius of Gyration	^a^ RMSF	^b^ SASA	^c^ H-Bonds			
					Intra	Prot-Solv	Prot-Mem	Mem-Solv
FAAH-r1	0.54 ± 0.03	3.38 ± 0.01	0.12 ± 0.10	461.30 ± 4.31	886 ± 14	1686 ± 24	50 ± 6	1497 ± 27
FAAH-r2	0.53 ± 0.03	3.33 ± 0.01	0.12 ± 0.11	456.15 ± 4.98	905 ± 14	1653 ± 24	41 ± 5	1525 ± 25
FAAH–macamide complex					Intra	Prot-maca	Solv-maca	
3-MBO-s1	0.22 ± 0.01	2.29 ± 0.01	0.10 ± 0.05	211.93 ± 3.02	402 ± 10	0.17 ± 0.38	0.96 ± 0.81	
3-MBL-s1	0.18 ± 0.01	2.28 ± 0.01	0.10 ± 0.05	212.83 ± 3.53	408 ± 10	0.13 ± 0.35	2.56 ± 1.18	
3-MBN-s1	0.21 ± 0.01	2.29 ± 0.00	0.10 ± 0.04	212.92 ± 2.74	415 ± 10	0.21 ± 0.42	3.37 ± 1.26	
3-MBO-s2	0.21 ± 0.01	2.29 ± 0.01	0.10 ± 0.05	213.56 ± 3.38	415 ± 10	1.02 ± 0.38	1.06 ± 0.82	
3-MBL-s2	0.23 ± 0.01	2.29 ± 0.00	0.11 ± 0.06	218.88 ± 2.95	412 ± 10	0.17 ± 0.38	0.68 ± 0.66	
3-MBN-s2	0.20 ± 0.02	2.29 ± 0.00	0.11 ± 0.06	215.71 ± 2.93	412 ± 9	0.09 ± 0.29	0.94 ± 0.77	

Values in *^a^* nanometers, *^b^* square nanometers, and *^c^* number of H-bonds formed. All values were obtained at the last 100 ns of the MD trajectories.

**Table 3:** Average MM/PBSA free energies of rFAAH–macamide complexes.

System	Van der Waals	Electrostatic	Polar Solvation	SASA	BFE
3-MBO-s2	−214.10 ± 0.90	−104.52 ± 1.38	126.82 ± 0.78	−26.98 ± 0.07	−218.77 ± 2.72
3-MBL-s2	−225.38 ± 0.93	−19.34 ± 1.23	62.30 ± 0.88	−26.24 ± 0.09	−208.66 ± 2.55
3-MBN-s2	−177.85 ± 1.24	−18.24 ± 1.34	46.68 ± 0.92	−23.44 ± 0.11	−172.85 ± 3.23
3-MBO-s3	−173.36 ± 0.93	−4.98 ± 0.64	68.21 ± 0.89	−22.14 ± 0.10	−132.27 ± 2.48
3-MBL-s3	−188.88 ± 1.19	−17.37 ± 1.31	109.81 ± 1.10	−22.20 ± 0.10	−118.64 ± 2.33
3-MBN-s3	−90.17 ± 1.95	−10.50 ± 1.01	38.27 ± 1.56	−13.46 ± 0.27	−75.85 ± 4.48

All values are given in kJ/mol.

**Table 4:** Per-residue analysis of the probability of the formation of potential binding pockets.

	Residue	FAAH				Site 2			Site 3		
		R1-chA	R1-chB	R2-chA	R2-chB	3-MBO	3-MBL	3-MBN	3-MBO	3-MBL	3-MBN
MAC	Asp403	0.21	0.60	0.36	0.61	0.11	0.05	0.10	0.38	0.19	0.15
	Ile407	0.35	0.65	0.46	0.78	0.11	0.06	0.22	0.05	0.17	0.05
	Arg486	0.13	0.37	0.25	0.27	0.39	0.34	0.46	0.34	0.32	0.32
	Ile530	0.15	0.33	0.21	0.37	0.03	0.06	0.02	0.02	0.08	0.01
ACB	Tyr335	0.31	0.81	0.25	0.31	0.54	0.57	0.69	0.13	0.20	0.53
	Glu373	0.20	0.77	0.26	0.34	0.09	0.21	0.24	0.07	0.03	0.07
	Arg428	0.30	0.46	0.13	0.21	0.55	0.45	0.76	0.16	0.38	0.74
	Phe527	0.18	0.45	0.05	0.02	0.17	0.18	0.33	0.04	0.09	0.23
MAC/ACB transition region	Phe381	0.70	0.61	0.66	0.90	0.86	0.49	0.81	0.57	0.69	0.89
	Phe432	0.35	0.98	0.63	0.60	0.73	0.65	0.68	0.63	0.79	0.61
	Trp531	0.22	0.66	0.24	0.29	0.58	0.37	0.77	0.16	0.34	0.60
Catalytic triad	Lys142	0.34	0.26	0.53	0.45	0.02	0.01	0.29	0.06	0.03	0.20
	Ser217	0.38	0.54	0.68	0.47	0.44	0.24	0.39	0.39	0.50	0.55
	Ser241	0.75	0.89	0.65	0.82	0.68	0.53	0.62	0.68	0.82	1.00
Oxyanion hole	Ile238	0.50	0.72	0.54	0.75	0.61	0.53	0.74	0.68	0.80	0.81
	Gly239	0.73	0.60	0.47	0.68	0.74	0.66	0.65	0.71	0.62	0.81
	Gly240	0.47	0.68	0.13	0.51	0.44	0.41	0.43	0.34	0.50	0.49

## Data Availability

The data presented and analyzed in this study are available from the corresponding author on reasonable request.

## Supplementary Materials

The following supporting information can be downloaded at: https://www.mdpi.com/article/10.3390/molecules30020333/s1, Figure S1: Electrostatic potential surfaces of the initial structures of the FAAH enzyme; Figure S2: Structural alignment of the initial and final configurations obtained from molecular dynamics calculations; Figure S3: Minimum energy structures of the rFAAH enzyme obtained in MD simulations; Figure S4: Electrostatic potential surfaces of the minimum energy structures of the rFAAH enzyme obtained in both replicates; Figure S5: Main interactions of macamides with rFAAH residues. Figures show the initial and final configurations of the MD trajectories; Table S1: Top 10 residues that contribute to the binding free energy in the rFAAH–macamide interaction.

## Abbreviations

The following abbreviations are used in this manuscript:

3-MBO	*N*-(3-methoxybenzyl)oleamide
3-MBL	*N*-(3-methoxybenzyl)linoleamide
3-MBN	*N*-(3-methoxybenzyl)linolenamide
rFAAH	rat fatty acid amide hydrolase
