# Supplementary material for: Anticonvulsant Effects of Synthetic N-(3-Methoxybenzyl)oleamide and N-(3-Methoxybenzyl)linoleamide Macamides: An In Silico and In Vivo Study
Source: Molecules. 2025 Jan 15;30(2):333. doi: 10.3390/molecules30020333 (PMC11767965; doi:10.3390/molecules30020333)
Supplement: Supplementary file 1 [file molecules-30-00333-s001.zip › molecules-3379807-supplementary.pdf]

Anticonvulsant Effects of Synthetic *N*-(3-Methoxybenzyl)oleamide and *N*-(3-Methoxybenzyl)linoleamide Macamides: An In Silico and In Vivo Study

January 15, 2025

Karin J. Vera-López[1‡], Jorge Alberto Aguilar-Pineda[2‡], Rodrigo Martín Moscoso-Palacios[1], Gonzalo Davila-Del-Carpio[1], José Luis Manrique-Murillo[3], Badhin Gómez[3], Minerva González-Melchor[2*], and Rita Nieto-Montesinos[1*].

[1]Escuela Profesional de Farmacía y Bioquímica, Universidad Católica de Santa María, Urb. San José s/n—Umacollo, Arequipa, 04000, Perú

[2]Instituto de Física "Luis Rivera Terrazas", Benemérita Universidad Autónoma de Puebla, Av San Claudio, Edificio IF-1, Ciudad Universitaria, Puebla, Pue. 72570, México

[3]Centro de Investigación en Ingeniería Molecular— CIIM, Universidad Católica de Santa María, Urb. San José s/n—Umacollo, Arequipa, 04000, Perú

Keywords: Macamides; Epilepsy; Neuroprotective effects; rFAAH; Molecular Dynamics.

*Authors to whom correspondence should be addressed.
e-mail: minerva@ifuap.buap.mx (M. González-Melchor); rnieto@ucsm.edu.pe (R. Nieto-Montesinos)

Supplementary Figures

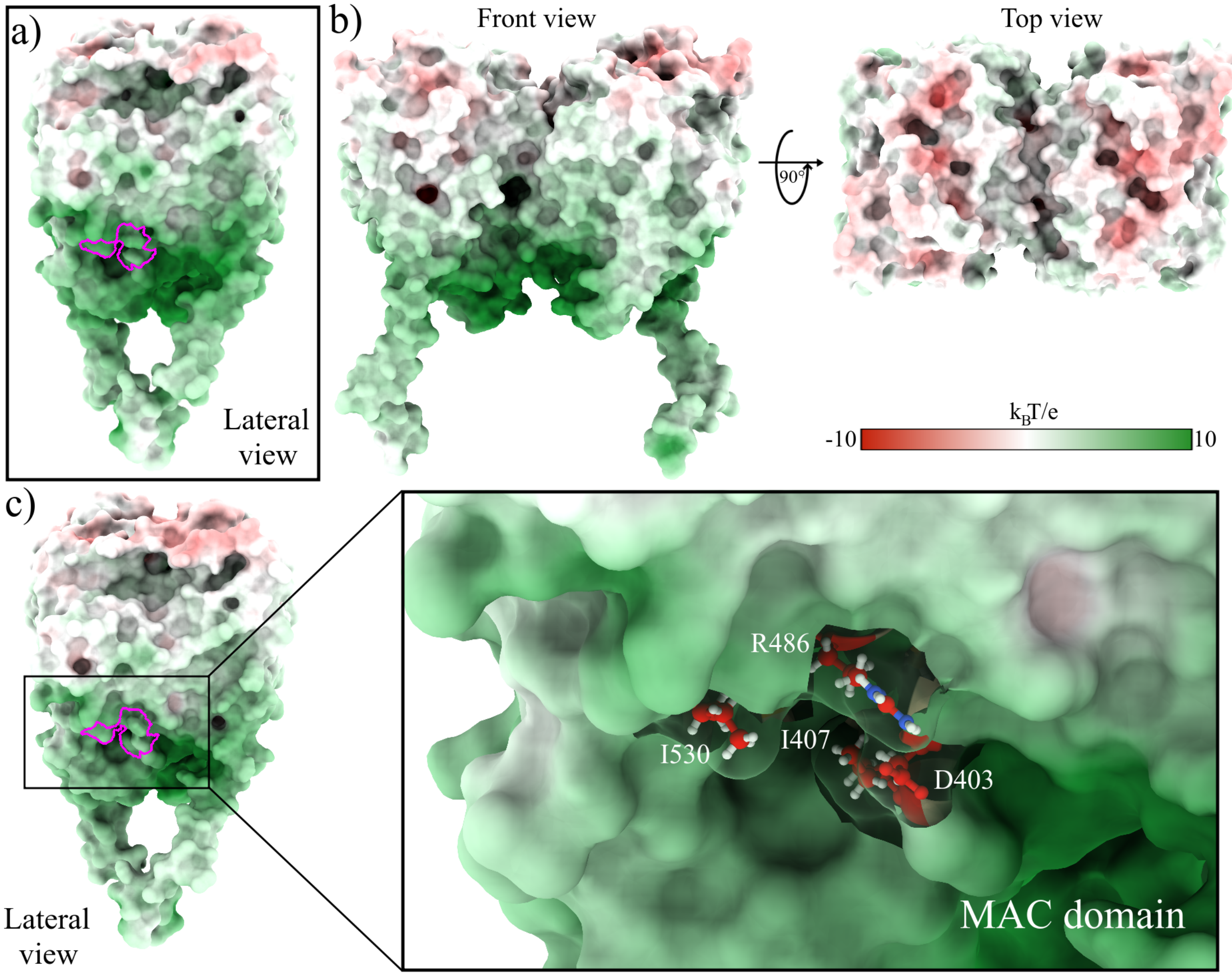

Figure S1. Electrostatic potential surfaces of the initial structures of the FAAH enzyme. (**a**) Side view of the structure of the first replica. The position of the amino acids that form the membrane access channel (MAC) is highlighted in magenta. (**b**) Front and top views of the second simulated replica. (**c**) Side view of the second replica. Close-up of amino acids D403, I407, R486 and I530. In all electrostatic surfaces, green color represents the electrophilic regions, red represents the nucleophilic regions, and white represents the hydrophobic regions.

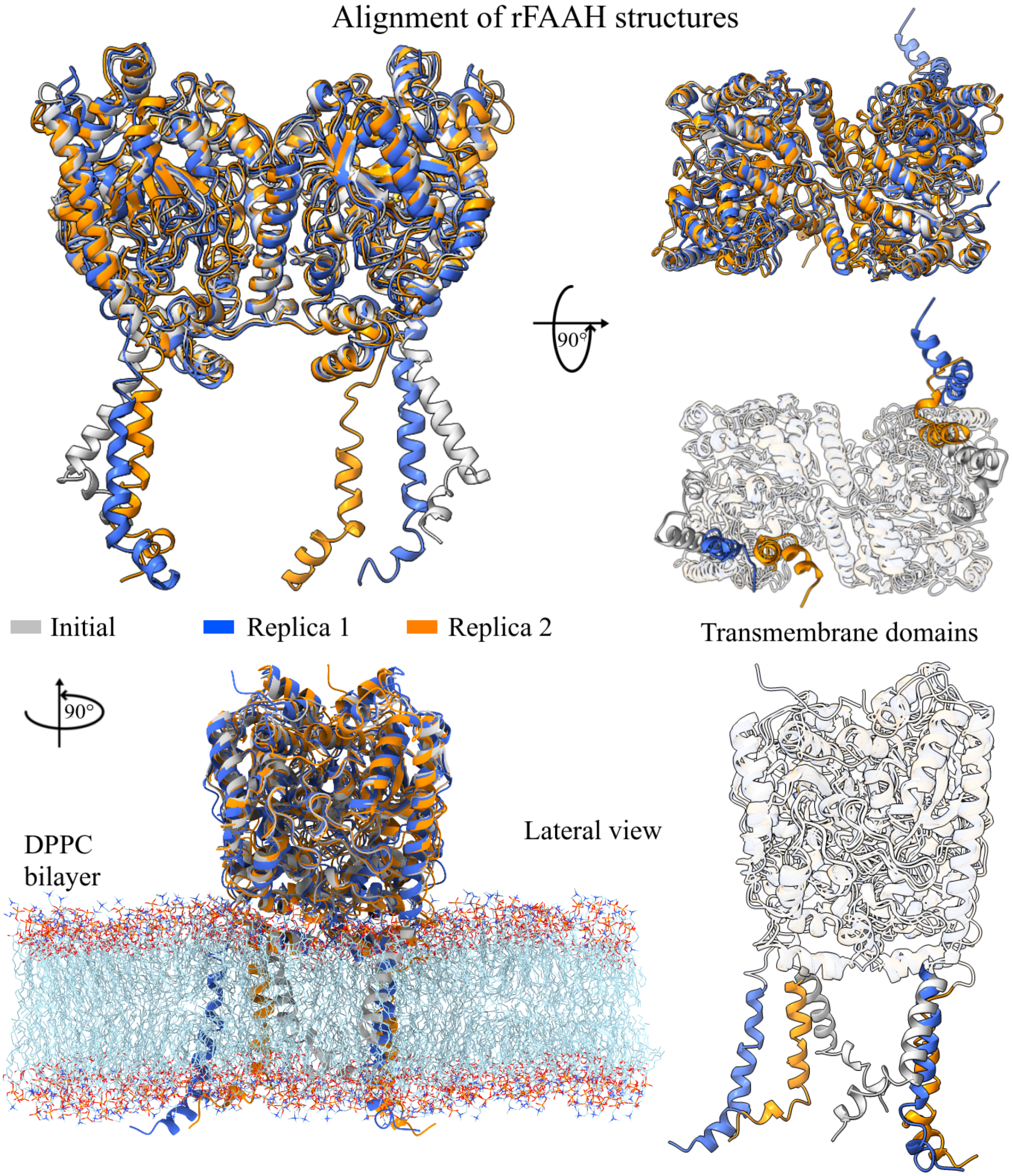

Figure S2. Structural alignment of the initial and final configurations obtained from molecular dynamics calculations. The structures show high conservation of both monomers' cytoplasmic (R30-D403, N434-S579) and intramembrane (L404-L433) domains. On the other hand, the transmembrane domains (T9-L29) showed higher mobility regarding the initial configuration.

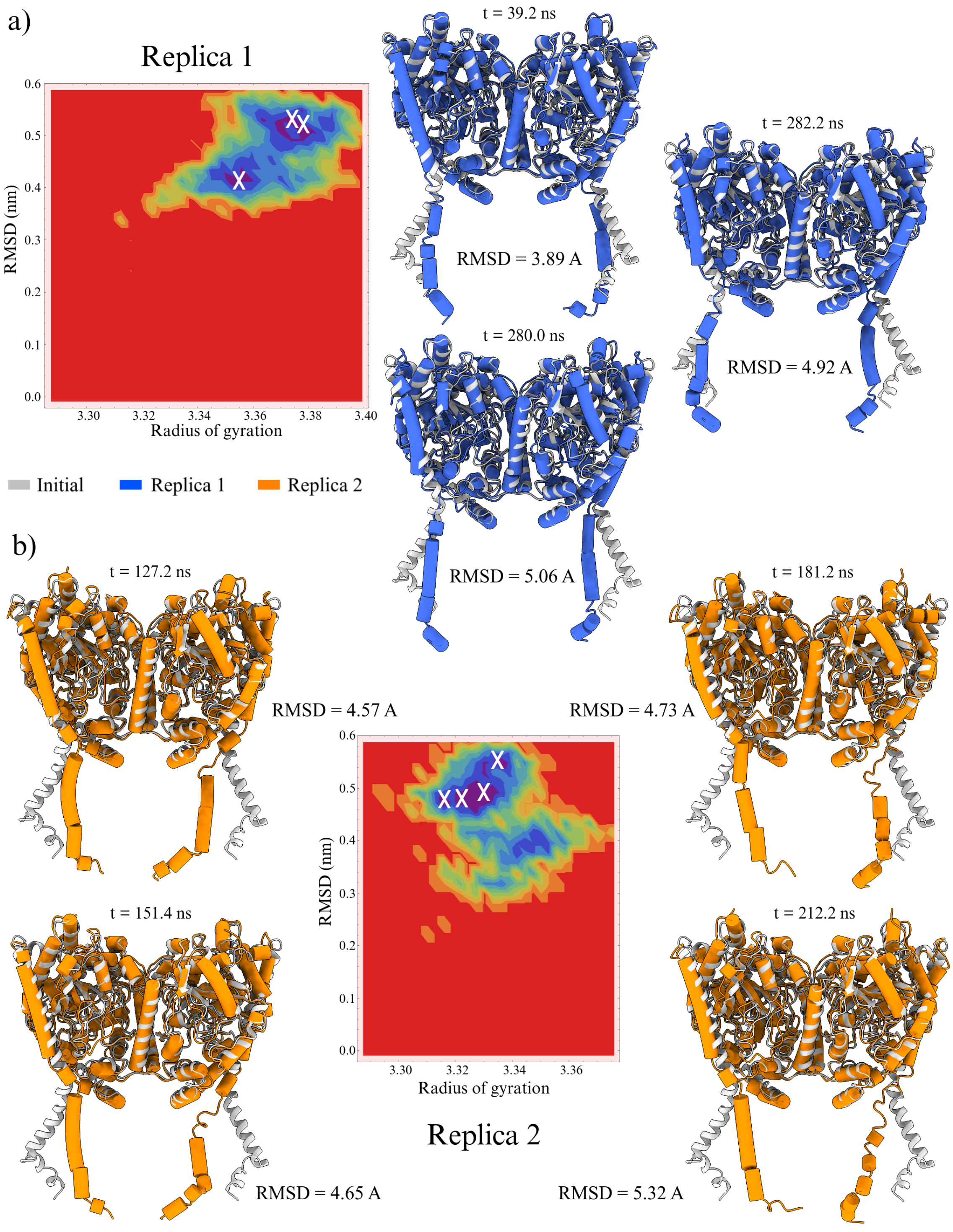

Figure S3. Minimum energy structures of the rFAAH enzyme obtained in MD simulations. (**a**) Replica 1. (**b**) Replica 2. The 2D heatmaps show the different energy substates of the configurations along the MD trajectories. The color range indicates high-energy (red) and low-energy (blue) states. The white marks indicate the energy minima corresponding to the 3D structures. The 3D representations are structurally aligned with the initial configuration and their obtained RMSD values.

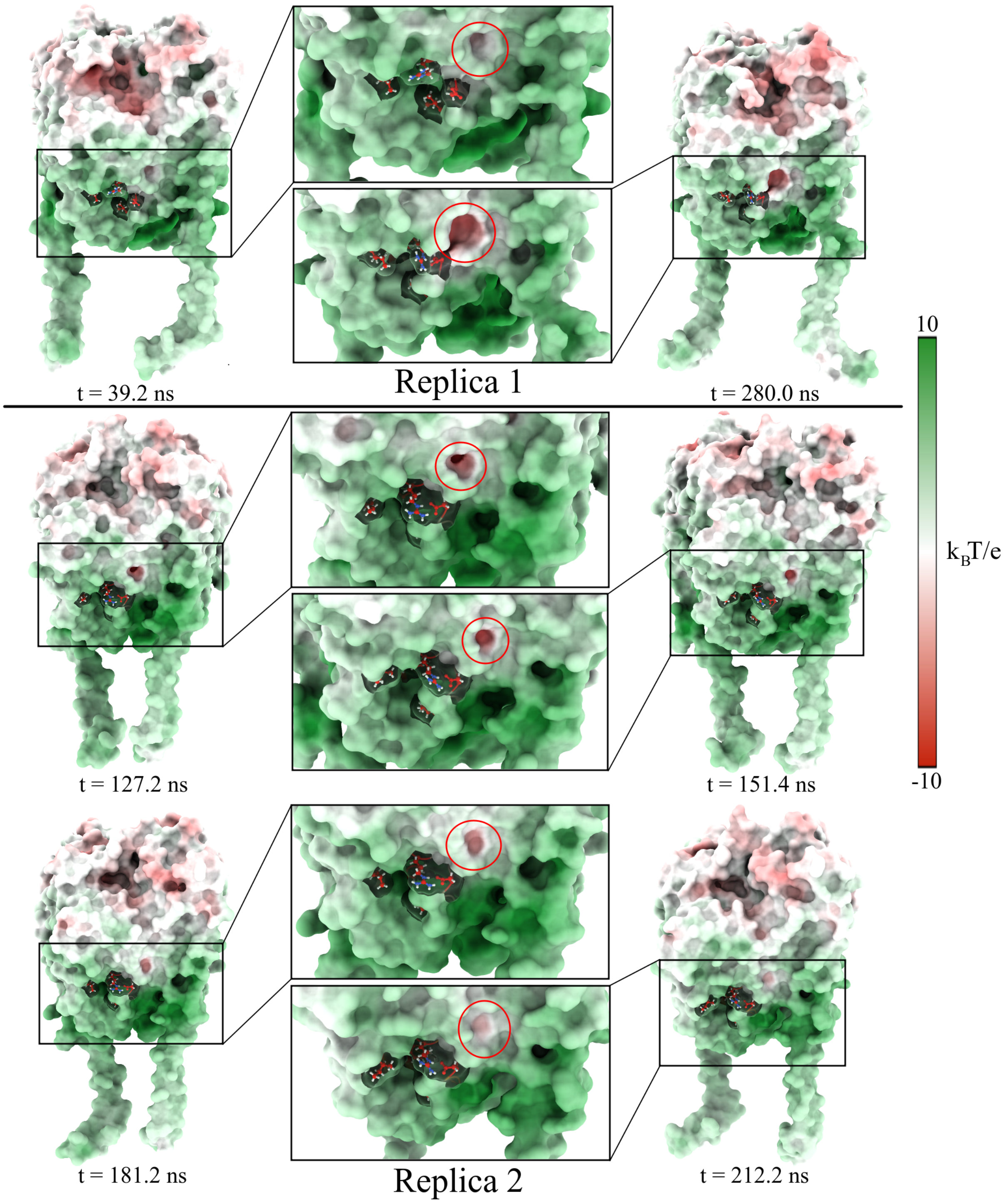

Figure S4. Electrostatic potential surfaces of the minimum energy structures of the rFAAH enzyme obtained in both replicates. The boxes show the region where the membrane access channel (MAC) and the nucleophilic pocket (NPC) formed by residues D195, S197, Q203, and N482 are located.

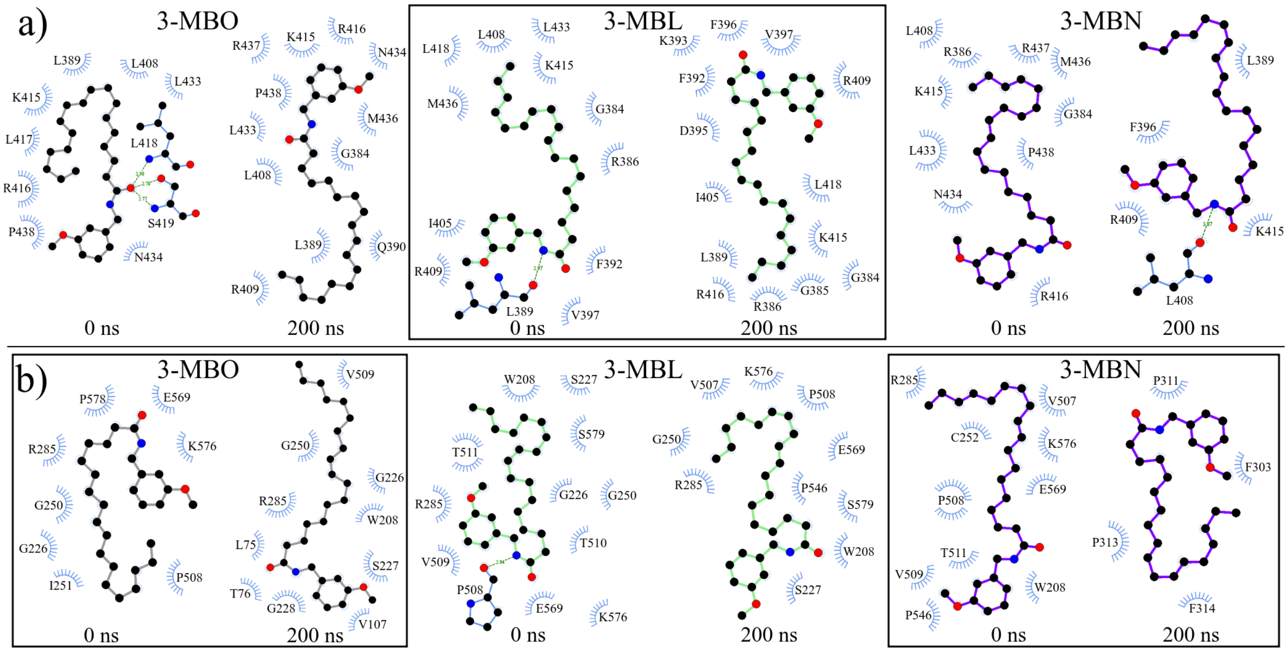

Figure S5. Main interactions of macamides with rFAAH residues. Figures (**a**,**b**) show the initial and final configurations of the MD trajectories.

Supplementary Table

Table S1. Top 10 residues that contribute to the binding free energy in the rFAAH–macamide interaction.

Site	Macamide		
	3-MBO	3-MBL	3-MBN
Site 2	L389 (**-110.7**), P438 (**-42.9**), R437 (**-42.5**), Q390 (**-34.9**), R386 (**-34.8**), M436 (**-32.4**), V397 (**-32.0**), N434 (**-18.8**), L433 (**-15.1**), F392 (**-14.4**)	R386 (**-50.8**), V397 (**-37.9**), L389 (**-28.5**), F388 (**-25.3**), I405 (**-24.2**), F392 (**-14.9**), G384 (**-12.6**), F396, (**-12.4**), K393 (**-11.0**), M436 (**-9.7**)	L389 (**-65.6**), M436 (**-38.6**), V397 (**-22.4**), F392 (**-19.0**), R386 (**-18.4**), P438 (**-17.3**), F388 (**-14.0**), L404 (**-11.8**), G384 (**-11.0**), I407 (**-7.5**)
Site 3	S227 (**-50.4**), W208 (**-40.7**), P207 (**-39.5**), P508 (**-24.5**), V107 (**-23.6**), G250 (**-15.2**), G110 (**-12.7**), G226 (**-8.4**), G228 (**-7.2**), P546 (**-6.5**)	P508 (**-41.2**), W208 (**-20.9**), G250 (**-14.2**), P546 (**-14.1**), V507 (**-10.4**), C252 (**-9.2**), T511 (**-8.7**), V509 (**-7.2**), R352 (**-6.5**), D286 (**-5.7**)	L522 (**-58.9**), M337 (**-23.6**), R343 (**-20.9**), L312 (**-20.3**), P311 (**-19.2**), P313 (**-15.2**), K524 (**-13.5**), P340 (**-13.1**), Y523 (**-12.3**), P310 (**-11.3**)
